# Hard Mating Aggregation as Evidence of Polyandry in the Red‐Tailed Boa, *Boa constrictor* (Squamata: Boidae), in a Brazilian Caatinga Population

**DOI:** 10.1002/ece3.71514

**Published:** 2025-05-28

**Authors:** Selma Maria de Almeida‐Santos, Renan Augusto Ramalho

**Affiliations:** ^1^ Laboratory of Ecology and Evolution Instituto Butantan São Paulo Brazil

**Keywords:** mating system, reproductive behavior, sexual size dimorphism, snake, xeric habitat

## Abstract

This study reports a reproductive aggregation of a female *Boa constrictor* with five males in the Brazilian Caatinga. The observation reinforces the evidence of a polyandrous system, favoring larger females that copulate with different males. Our record highlights the influence of sexual size dimorphism in the formation of aggregations.

## Graphical abstract

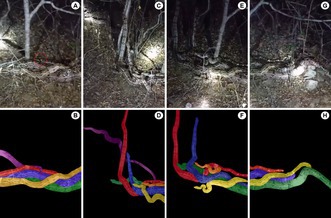

## RESUMO

Este estudo relata uma agregação reprodutiva de uma *Boa constrictor* fêmea com cinco machos na Caatinga brasileira. A observação reforça a evidência de um sistema poliândrico, favorecendo fêmeas maiores que copulam com machos diferentes. Nosso registro destaca a influência do dimorfismo sexual de tamanho na formação de agregações.

## 1 Introduction

A mating system is defined based on the behavioral strategy adopted to obtain sexual partners (Emlen and Oring 1977). These systems can vary according to the ability of one sex to monopolize members of the opposite sex, the availability of partners, and the spatial distribution of resources (Emlen and Oring 1977; Rivas and Burghardt 2005). Over the past 20 years, evidence has suggested that the most common mating system in snakes is polygynandry (Rivas and Burghardt 2005; Schuett et al. 2019; Almeida‐Santos and Sawaya 2022), but these systems may vary between populations and taxonomic groups (Duvall et al. 1993; Rivas and Burghardt 2005; Schuett et al. 2019).

Some species of boids and pythonids are polyandrous (Rivas and Burghardt 2005; Schuett et al. 2019). Previous studies showed that, as a “capital breeder”, female boas accumulate energy for a long period before initiating reproductive investment (Bertona and Chiaraviglio 2003; Cardozo and Chiaraviglio 2008), preventing them from reproducing annually and decreasing the ratio of receptive females per male during the reproductive season (male‐biased OSR, see Rivas and Burghardt 2005). This increases the potential for females to mate multiple times and reduces the potential for males, who tend to choose larger and more attractive females (Rivas and Burghardt 2005).

One of the main behavioral components of the polyandrous system is reproductive aggregations that can last for several days and, in the best‐known example in South American anacondas, consist of 2–13 males coiled around a single female, courting and attempting to mate, tending to stay close to her until the end of the attractiveness period (Rivas and Burghardt 2001, 2005). In the last few years, studies have described many examples of this behavior in different snake species such as *Python molurus* (Bartoszek et al. 2018), *Liasis fuscus* (Brusch IV et al. 2019), *Dipsas alternans* (Marinho et al. 2020), *Philodryas olfersii* (Banci et al. 2021), *Dromicodryas bernieri* and *D. quadrilineatus* (Fukuyama et al. 2022), *Vipera ammodytes* (Čubrić and Crnobrnja‐Isailović 2023), and *Philothamnus natalensis* (Kyle and Downs 2025).

Reproductive aggregations in the genus *Boa* have been reported in *B. occidentalis* (Bertona and Chiaraviglio 2003), *B. imperator* (Schuett et al. 2019) and even in *B. constrictor* (Almeida‐Santos and Sawaya 2022). However, in all the records mentioned, only one female engaged with up to three males was reported. These aggregations appear to be related to the female‐biased sexual size dimorphism (Shine 1994; Rivas and Burghardt 2005), where males position themselves above the female's body while courting her and try to intertwine their tails, with no registry of aggressive behavior (e.g., biting) between rivals (Schuett et al. 2019).

The red‐tailed boa, *Boa constrictor* , is widely distributed in South America (Uetz et al. 2024). This large semi‐arboreal viviparous snake exhibits female‐biased sexual size dimorphism and a seasonal reproductive cycle (Pizzatto and Marques 2007; Nogueira et al. 2019; Garcia and de Almeida‐Santos 2022; Ryerson and Goulet 2021; Ryerson et al. 2022). In Brazilian populations, reproductive aggregation occurs in the austral winter (dry season), associated with increased gonadal activity (vitellogenesis and spermatogenesis) in males and females (Garcia and de Almeida‐Santos 2022; Almeida‐Santos and Sawaya 2022), which may remain aggregated for several days (Almeida‐Santos and Sawaya 2022). This study shows a hard mating aggregation as evidence of polyandry in *B. constrictor* .

## 2 Methods

The event took place on 26 July 2024 (austral winter) around 2200 in an area of Caatinga, when a group of observers came across six *Boa constrictor* in the surroundings of Serra Talhada municipality (−7.89409, −38.20812), Pernambuco state, northeastern Brazil. The description was based on this fortuitous encounter, which was videotaped. We identified individuals based on their behaviors and physical attributes (e.g., size, robustness, pholidosis), and captured frames of the footage to illustrate the behaviors performed during aggregation.

**FIGURE 1:**
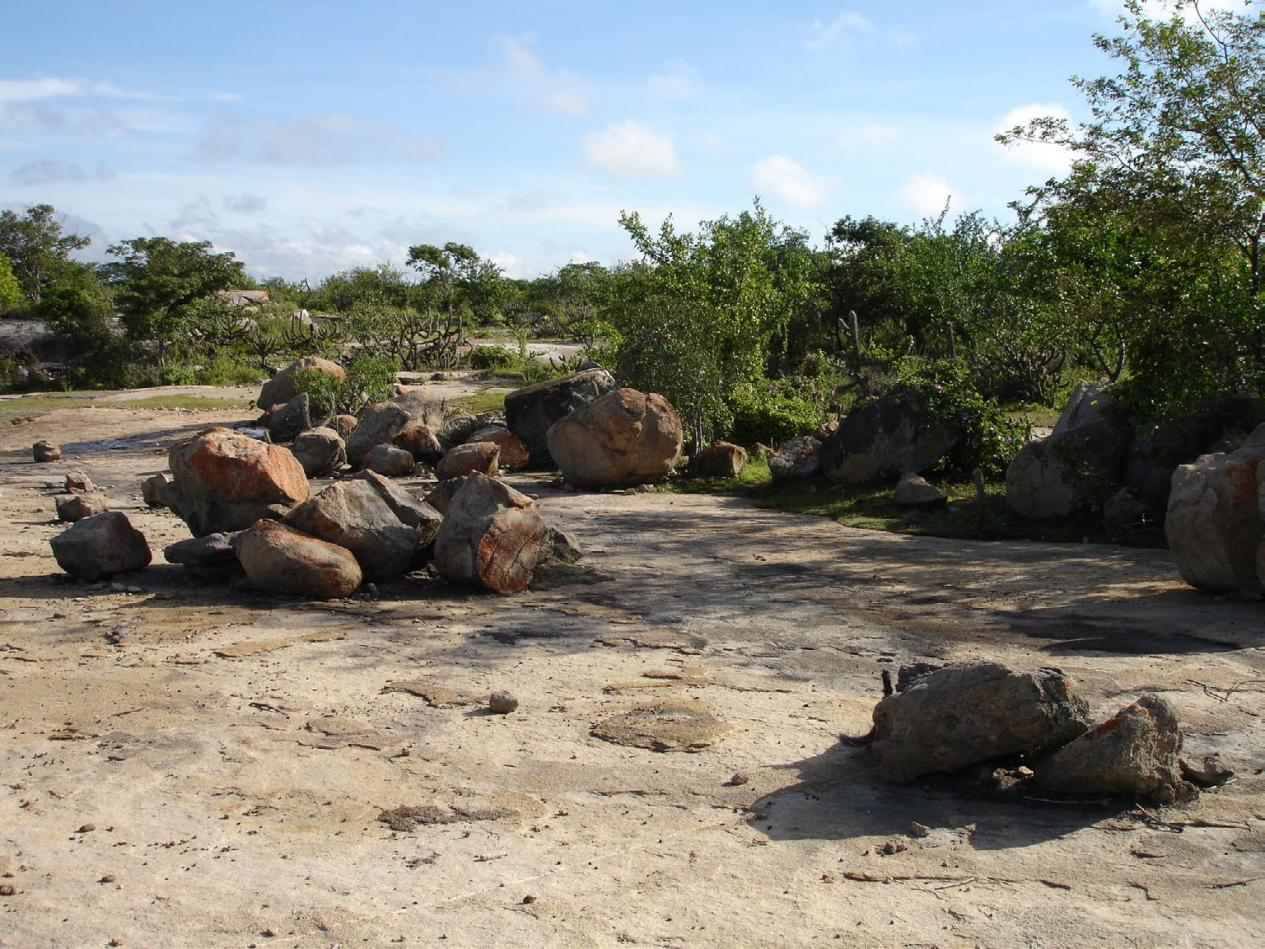
Caatinga area in the municipality of Parnamirim, Pernambuco state, northeast Brazil, 150 km from Serra Talhada.

The predominant climate in this area is semi‐arid (Alvares et al. 2013), with low annual rainfall (250–900 mm) and high incidence of solar radiation that makes average temperatures high throughout the year (25°C–30°C) (Alves et al. 2009; Moro et al. 2016), resulting in long periods of drought between June and December (Moro et al. 2016). The vegetation is adapted to the water deficit, with predominance of shrubs and small deciduous trees with superficial roots, and cacti typical of this xeric habitat (Fernandes and Queiroz 2018) (Figure 1).

## 3 Results

Based on the photographs, the snake's behavior and its body sizes, the six aggregated individuals corresponded to five males (total length *ca*. 1.2–1.5 m) and one female (total length *ca*. 2.0–2.5 m).

**FIGURE 2:**
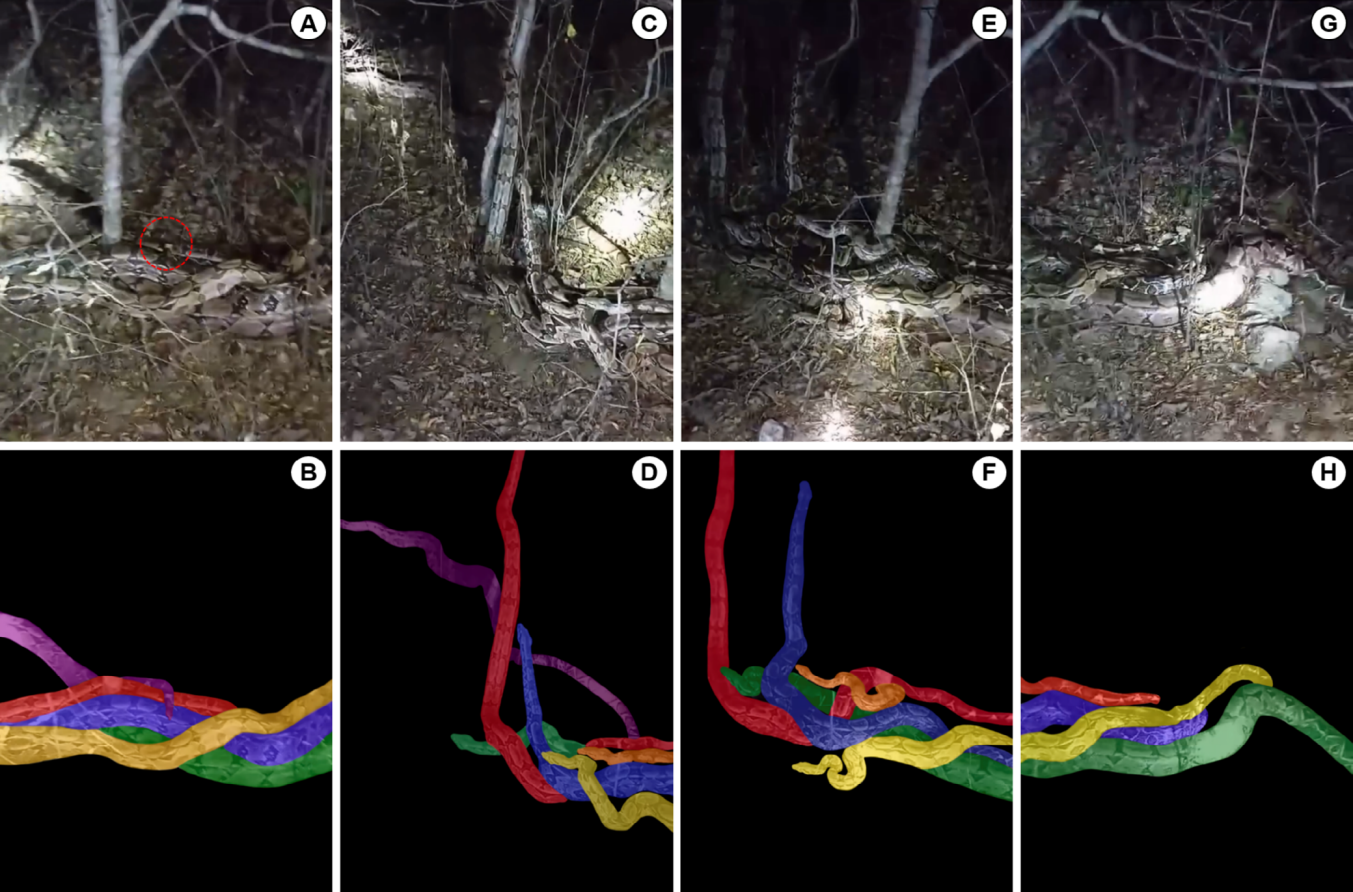
Reproductive aggregation of *Boa constrictor* showing males #1 (purple), #2 (red), #3 (blue), #4 (yellow), #5 (orange) and a female (green). (A, B) Male #1 leaving the aggregation (red circle indicates the male's tail) and males #2, #3, and #4 aligned with the female's body. (C, D) Male #2 climbing the trunk of a tree followed by male #3, while males #4 and #5 remained courting the female. (E, F) Female with her body stretched out and pressed to the ground with the four males on her back. (G, H) Tails of males #3, #4 and #5 (tail positioned behind the other individuals) aligned with the female's body.

When spotted, the six snakes involved in the reproductive aggregation began to disperse due to the loud noise and strong flashlights coming from the observers. At the time they were observed, a large male (male #1) had his tail wrapped around the female's tail, but quickly it was possible to observe the tail of this male leaving the other individuals (males #2, #3 and #4) aligned with the female's body (Figure 2A,B). Feeling threatened by the human presence, male #2 raised the anterior region of his body and started to climb the trunk of a tree (*Aspidosperma populifolium*), followed by male #3, who also made the same movement while displaying intense tongue flicking (Figure 2C,D). Males #4 and #5 remained aligned over the female's body, apparently initiating tail search in a copulatory attempt (Figure 2C,D). The female remained with her body stretched out and pressed to the ground with the four males on her back, and was only able to move her head when males #2 and #3 moved towards the tree trunk (Figure 2E,F). The tails of the males #3, #4 and #5 remained aligned with the female to wrap them around hers and oppose their cloaca to copulate (Figure 2G,H). It was not possible to observe the eversion and insertion of the hemipenis of any of the males into the female's cloaca. Also, no aggression was observed between the individuals.

## 4 Discussion

Our observation is the first to report a reproductive aggregation with such a high ratio of males to a single female in the genus *Boa*. Bertona and Chiaraviglio (2003) showed that *B. occidentalis* can exhibit aggregations composed of one female with up to three males. Schuett et al. (2019) also made a similar record in *B. imperator* , and Almeida‐Santos and Sawaya (2022) observed a female *B. constrictor* aggregating with two other males. Information on the reproductive behaviors of this group remains scarce, but recent observations (Schuett et al. 2019; Almeida‐Santos and Sawaya 2022; this work) show that boa aggregations, in some ways, resemble *Eunectes murinus* aggregations, both for the long period of time in which individuals remain aggregated and for the high number of participants (Rivas and Burghardt 2005; Schuett et al. 2019; Almeida‐Santos and Sawaya 2022).

During courting, *Eunectes murinus* males typically coil themselves around the posterior portion of a female's body, although they may occasionally embrace her entire length (Rivas et al. 2007). As they coil, males use their spurs to scratch the partner (Rivas et al. 2007). Otherwise, in *Boa constrictor* , the female remains fully stretched out on the ground while males climb onto their backs, aligning their bodies (Almeida‐Santos and Sawaya 2022; Anzai et al. 2023). Then, the male gently rubs its spurs along the sides of the female's body in a posterior‐to‐anterior motion, encouraging the female to adjust their position. This behavior facilitates cloacal alignment and the insertion of the hemipenis (Anzai et al. 2023).

Snakes appear to exhibit a relationship between sexual size dimorphism and the mating system adopted (Shine 1994). Among boids, male and female *Epicrates* exhibit minimal size differences and engage in male–male combats during the reproductive season, characteristics of a polygynous system (Pizzatto et al. 2006; Pizzatto and Marques 2007; Guedes et al. 2019; Almeida‐Santos et al. 2024). On the other hand, females of the genera *Boa* and *Eunectes* are significantly larger than males and aggregate during the reproductive season, closer to a polyandrous system (Bertona and Chiaraviglio 2003; Rivas and Burghardt 2005; Pizzatto and Marques 2007).

Bertona and Chiaraviglio (2003) described the mating system of *Boa occidentalis* as “prolonged mate searching polygyny”. In this system, males search for widely distributed and scarce receptive females, so sexual selection should act on mate searching ability and not male–male combat (Duvall et al. 1992). However, Rivas and Burghardt (2005) proposed a new hypothesis that contradicts this classification. Saying that the system is polygynous implies that males have a greater potential to obtain more mates than females. However, the opposite was observed, since females monopolized several males. Other evidence supports the occurrence of polyandry among some boid species: (1) females do not usually reproduce every year due to the difficulty in recovering from the high reproductive investment, leading to a male‐biased OSR (Bertona and Chiaraviglio 2003; Rivas and Burghardt 2005), where the low supply of reproductive females per season implies (2) high expenditure of time and energy by males during the search for mates, increasing their mortality rates (Rivas and Burghardt 2005). The male‐biased OSR also leads (3) males to select females with better fertility indicators (Rivas and Burghardt 2005) and (4) favors a scenario where the female copulates with several males, but the opposite does not seem to occur, since males tend to remain entwined with the female for long periods in the aggregation (Rivas and Burghardt 2005; Schuett et al. 2019; Almeida‐Santos and Sawaya 2022).

To locate receptive females scattered in the environment, males need to orient themselves using chemical cues (Halpern 1992; Bertona and Chiaraviglio 2003). The ability to recognize conspecifics through pheromones plays an essential role in the formation of reproductive aggregations and indirectly affects reproductive success (Lemaster et al. 2001; Bertona and Chiaraviglio 2003). It is known that males of *Boa occidentalis* and *Epicrates alvarezi* respond differently to being stimulated with male and female skin, showing a stronger response in the second scenario (Briguera et al. 1997; Chiaraviglio and Briguera 2001), and that males of *Morelia spilota* are very accurate in following trails during the reproductive season, when activity levels are high (Slip and Shine 1988).

In *Boa constrictor* , vitellogenesis and spermatogenesis begin in the austral autumn and peak during winter (both dry seasons) (Bento et al. 2019; Pizzatto and Marques 2007). Most recorded mating of this species occurred during winter, associated with gonadal activity of both males and females (Bento et al. 2019; Pizzatto and Marques 2007, Garcia and de Almeida‐Santos 2022). Thus, our aggregation record coincides not only with other reported copulations, but also with the aggregation observed in August by Almeida‐Santos and Sawaya (2022).

It is interesting to note that even under the adverse conditions of the dry season in the semi‐arid climate, the timing of the reproductive aggregation described here agrees with another similar observation made in the Brazilian Cerrado (Almeida‐Santos and Sawaya 2022), and with its South American congener *B. occidentalis* in the Argentine Chaco (Bertona and Chiaraviglio 2003). This may suggest that the reproductive cycle varies little between populations or species, but further studies on reproductive tactics, including histological techniques, are needed to test this hypothesis.

## 5 Conclusion

Our observation provides new information about reproductive behaviors that are rarely observed in wildlife snakes. Although reproductive aggregations have already been reported in the *Boa* genus, the number of individuals involved in our record is noteworthy, since the high ratio of males to one female reinforces the hypothesis that the group adopts a polyandrous system. However, new studies on its reproductive biology may contribute to the understanding of some factors that remain unknown, such as the occurrence of sperm storage in the oviduct, rates of multiple paternity, and how this may influence reproductive success.

## Author Contributions

**Selma Maria de Almeida‐Santos:** conceptualization (lead), data curation (equal), funding acquisition (lead), investigation (equal), methodology (equal), supervision (lead), validation (lead), visualization (equal), writing – original draft (equal), writing – review and editing (equal). **Renan Augusto Ramalho:** conceptualization (equal), data curation (equal), investigation (equal), methodology (equal), validation (equal), visualization (equal), writing – original draft (equal), writing – review and editing (equal).

## Conflicts of Interest

The authors declare no conflicts of interest.

## Data Availability

Data sharing is not applicable as no data were created or analyzed in this study.
